# AGING SUPPORT, HEALTH, AND WELL-BEING OF THE ELDERLY IN CHINA AND JAPAN

**DOI:** 10.1093/geroni/igz038.2241

**Published:** 2019-11-08

**Authors:** Emma Zang, Yuan Zhang

**Affiliations:** 1 Duke University, Durham, North Carolina, United States; 2 University of North Carolina at Chapel Hill, Chapel Hill, North Carolina, United States

## Abstract

Countries in East Asia have the largest aging population in the world. The consequences of aging largely depend on whether it is accompanied by a healthy, active, and high-quality life. This symposium aims to gain a better understanding of aging support and determinants of health in the contexts of two major East Asian countries - China and Japan. We will present new research using data from the Fukui Longitudinal Caregiver Study (FLCS) in Japan, and two most important aging surveys in China – the China Health and Retirement Longitudinal Study (CHARLS) and the Chinese Longitudinal Healthy Longevity Survey (CLHLS), addressing critical topics including retirement, family care, social mobility, and mortality. Song and Smith investigate the impact of hukou change on mental health in later life. Zang examines the effect of a man’s retirement on his wife’s mental and physical health in China. Zhang et al. explore the determinants of mortality in China by conducting a comprehensive analysis of life-course conditions, community characteristics, biological and physical functioning, and disease burden. Zeng et al. compare demographic, socioeconomic, behavioral characteristics and health phenotypes of centenarians in China and Italy. Wakui et al. focus on the emergence of compound caregiving and the relationship of caregiving status to burden, depression, and social support in Japan. The cross-national comparisons will be informative regarding aging in various contexts. We will discuss the potential for further investigations using population-based aging data from different countries.

